# A Rare Neonatal Infection: Methicillin-Resistant Staphylococcus aureus (MRSA) Orbital Cellulitis

**DOI:** 10.7759/cureus.65586

**Published:** 2024-07-28

**Authors:** Elizabeth Davis, Coral Molina, Adarsh Kancharla, Jaime Brown

**Affiliations:** 1 Family Medicine, Spartanburg Regional, Spartanburg, USA; 2 Pediatrics, Spartanburg Regional, Spartanburg, USA

**Keywords:** acute sinusitis, neonatal sepsis, methicillin resistant staphylococcus aureus bacteremia, ocular proptosis, subperiosteal abscess, febrile infant, comprehensive physical exam, methicillin resistant staphylococcus aureus (mrsa), late onset neonatal sepsis, preseptal orbital cellulitis

## Abstract

We present a case of orbital cellulitis as a rare cause of late-onset neonatal sepsis in a 4-week-old infant. Although ample literature describes orbital cellulitis in pediatric age groups, community-acquired orbital cellulitis as a cause of late-onset neonatal sepsis has not been well-described. We report one case of late neonatal sepsis in which orbital cellulitis and subsequent methicillin-resistant *Staphylococcus aureus *(MRSA) bacteremia were found. This case emphasizes the importance of early recognition of orbital cellulitis in neonates and awareness of this unique cause of sepsis.

## Introduction

Neonatal sepsis is divided into early- and late-onset categories, depending on the age of onset. Late-onset neonatal sepsis is defined as sepsis that occurs between days 7-60 of life [1] and is related to exposure during the birth process or postnatal exposure [2]. The presentation of neonatal sepsis can be challenging due to the wide range of causes and the lack of specificity at presentation. Some neonates with sepsis present with concerning symptoms such as respiratory distress or lethargy, but many may present with only vague symptoms such as temperature instability or increased irritability. When neonatal sepsis is suspected at presentation, evaluation of the blood, urine, and skin for a source of infection is necessary. This investigation also may require the evaluation of the cerebrospinal fluid (CSF), depending on the clinical scenario. Inflammatory markers, blood cultures, urinalysis, urine culture, and CSF studies when appropriate have a high sensitivity and specificity in diagnosing most serious bacterial infections, but many of these take time to result. In a neonate with suspected sepsis, it is imperative to initiate early empiric antibiotics to treat the suspected infection while awaiting completion of the work-up [3, 4].

In the United States, *Escherichia coli* is the most prevalent cause of neonatal sepsis in full-term neonates presenting with late-onset sepsis, followed by group B Streptococcus, *Streptococcus viridans*, and *Staphylococcus aureus *[2]. The incidence of late-onset neonatal sepsis for full-term non-ill-appearing infants presenting with fever is approximately 1.8% [5, 6]. Of those positive blood cultures, about six percent are caused by *Staphylococcus aureus* [2] with approximately 28% of those isolates being methicillin-resistant *Staphylococcus aureus* (MRSA) [3]. The mortality rate of infants infected with MRSA bacteremia or meningitis is 25%. Still, there is no difference between the mortality, morbidity, or length of hospital stay between MRSA and methicillin-sensitive *Staphylococcus aureus* (MSSA) infections within these patients [7].

Orbital cellulitis is inflammation caused by an infection of the muscle and fat posterior to the ocular septum and can be associated with severe complications [8]. The presentation of orbital cellulitis usually involves periocular edema, erythema, proptosis, and ophthalmoplegia, and is often associated with fever and leukocytosis [8]. Diagnosis can be made by these physical exam findings, with ophthalmoplegia being the most specific finding but not always present at presentation. Confirmation can be made via computed tomography (CT) imaging when there is uncertainty surrounding the diagnosis. Complications of orbital cellulitis arise when the infection spreads to the anatomical surroundings of the orbit and can lead to intracranial abscesses, subperiosteal abscesses, orbital abscesses, meningitis, and blindness [9]. 

Orbital cellulitis is most common in elementary school-aged children and often results from an upper respiratory tract infection, most commonly sinusitis. The proposed mechanism for why orbital cellulitis is more common in the pediatric age group than in adults is due to immaturity in immune system development [9] and vulnerabilities in the local structures between the preseptal space and sinus cavities [10]. The most common sinus cavity involved in sinusitis leading to orbital cellulitis is the ethmoid sinus, often with associated maxillary sinus involvement [11]. A common complication evaluated by CT imaging for orbital cellulitis is the development of a subperiosteal abscess. This development is a severe complication and can lead to vision loss, intracranial abscess development, or meningitis if left untreated [12, 13]. The most common pathogens in orbital cellulitis are staphylococcus aureus and streptococcus species, but aspirates are usually polymicrobial [9]. 

Empiric antibiotics for orbital cellulitis should cover upper respiratory tract microbes including MRSA and gram-negative bacteria. Additionally, until intracranial extension can be ruled out or if cellulitis is suspected to be secondary to chronic sinusitis or from an odontogenic source, antibiotic coverage should be expanded to cover anaerobes. Common empiric antibiotic regimens include vancomycin plus either ceftriaxone or cefotaxime to cover for MRSA and gram-negative pathogens. Anaerobic coverage can include the addition of metronidazole to the above regimen or can be covered by using a combination of vancomycin and ampicillin-sulbactam [13-14].

The use of corticosteroids and their effectiveness in this situation is not well established. There is conflicting evidence as to whether or not they are beneficial in leading to shorter hospital stays and reduced time to resolution or if they mask signs of worsening infection and lead to more operations and readmissions [15-17]. The addition of corticosteroids is usually up to the consulting ophthalmologist or otolaryngologist.

## Case presentation

A four-week-old full-term male born to a Group-B-Streptococcus-positive mother with adequate perinatal antibiotic treatment presented to a pediatrician for a routine well-child check and was noted at the time to have a fever. The patient’s mother noted that he had been more irritable for the 48-72 hours leading up to his appointment but attributed this to “constipation”. She denied any rhinorrhea, congestion, fevers, or cough at home but that day she noticed the skin around one eye to be mildly pink.

On admission, vital signs revealed a febrile temperature of 102.8F, a heart rate of 182 beats per minute, blood pressure of 99/48 mmHg, a respiratory rate of 38 breaths per minute, and an oxygen saturation of 98% on room air. On physical exam, the patient was noted to be ill-appearing and irritable. He had very mild right-sided periorbital swelling and a systolic flow murmur, and he was hypotonic on exam. There was no nuchal rigidity, nasal discharge, increased work of breathing, abnormal sounds on lung auscultation, or other skin changes. Extra-ocular movement appeared to be intact and there was no scleral injection, ocular discharge, or proptosis. Laboratory investigation was significant for an elevated C-reactive protein of 6.1 mg/dL (normal range 0.3-1.0 mg/dL), procalcitonin of 0.76 ng/mL (normal value <0.1 ng/mL), and absolute neutrophil count was 6,100 cells/mcL (normal range 2,500-6,000 cell/mcL). Cerebrospinal fluid (CSF) cytology, CSF culture, BioFire FilmArray Meningitis/Encephalitis Panel, BioFire Respiratory Pathogen 2.1 Panel (BioFire Diagnostics, Salt Lake City, USA), and urinalysis were negative for signs of infection. The patient was empirically placed on ceftriaxone and ampicillin on admission after blood cultures had been drawn.

**Figure 1:**
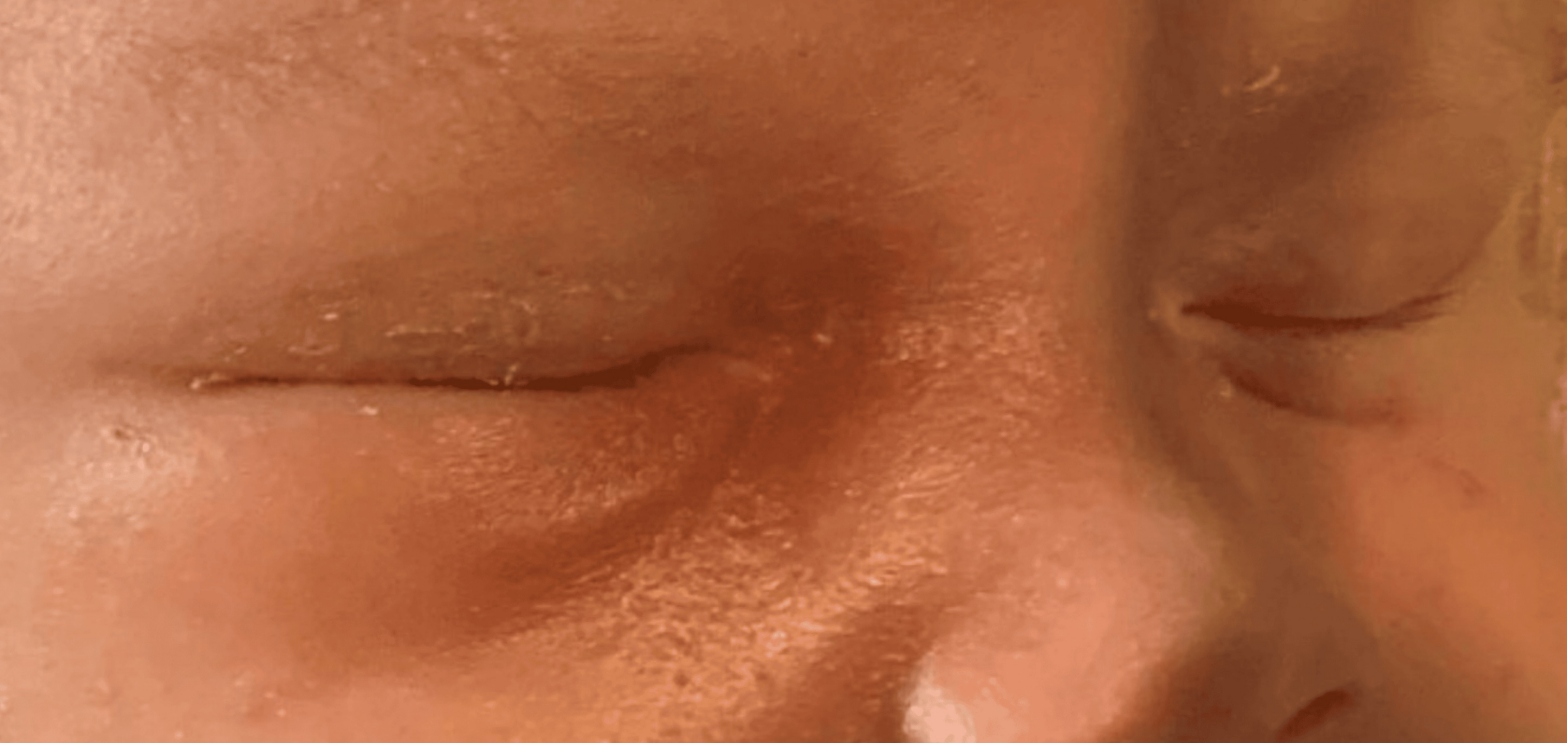
Right periorbital swelling and erythema noted on the night of admission

**Figure 2:**
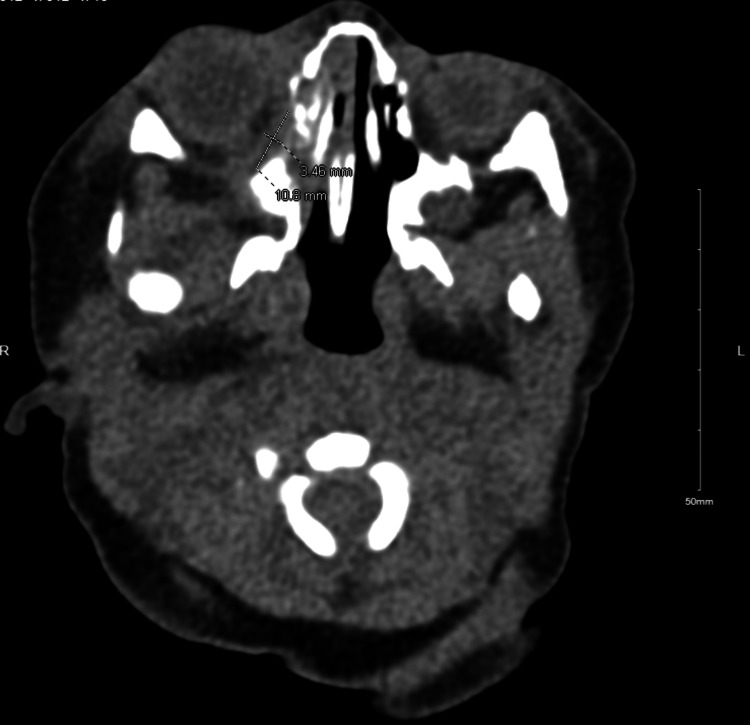
CT Scan Orbits/Sella with contrast shows lentiform collection along the right inferomedial extraconal orbit and associated trace right proptosis

On the night of admission, the right periorbital swelling was slightly more notable and slightly more erythematous (Figure 1). A computed tomography (CT) scan of the orbits was performed and the patient’s antibiotic coverage was broadened to include MRSA coverage with vancomycin and gram-negative anaerobic coverage with ampicillin/sulbactam. CT scan of orbits revealed a lentiform collection along the right inferomedial extraconal orbit suspicious for a developing subperiosteal abscess and associated trace right proptosis (Figure 2). Additionally, there was associated right paranasal sinus disease with complete opacification of the maxillary and ethmoid sinuses. An otolaryngologist was consulted and recommended adding nasal saline and intravenous dexamethasone for 48 hours for the treatment of orbital cellulitis. His blood culture grew gram-positive cocci in pairs and clusters at 24 hours incubation, ultimately identified as MRSA bacteria.

**Figure 3:**
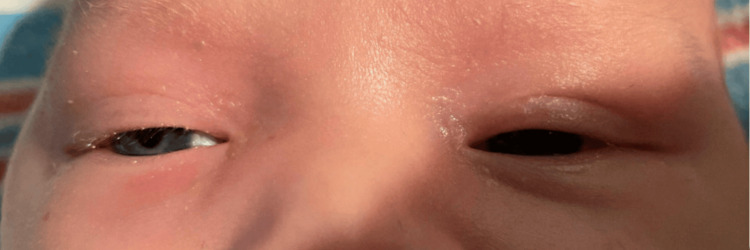
Right periorbital swelling and erythema much improved after 24 hours of treatment with vancomycin, ampicillin/sulbactam, and dexamethasone

On this treatment regimen, the periorbital swelling greatly improved after 24 hours (Figure 3). After MRSA susceptibilities returned, repeat blood cultures were negative for 48 hours, and the patient’s family was provided thorough education on antibiotic administration, the patient was discharged home on oral clindamycin for a total of 14 days of antibiotic treatment. He continued to do well with no recurrence or sequela from this infection thus far.

## Discussion

The differential diagnosis for an ill-appearing febrile infant includes bacterial and viral infections, trauma, allergic reactions, and immune reactions [18] and the work-up for each of these etiologies can vary significantly. In a four-week-old with irritability, fever, ill appearance, and poor tone, an infectious workup has to be pursued to rule out any life-threatening bacterial illnesses. The elevated C-reactive protein (CRP), procalcitonin, and absolute neutrophil count were concerning for a bacterial process underlying this particular infant's symptoms. Although orbital cellulitis is more common in children than adults, it is very rare in neonates and infants [19]. Despite its rarity, given the localization of an abnormal physical exam to this ill-appearing infant's eye, imaging was pursued and provided a diagnosis of orbital cellulitis. Empiric antibiotics for orbital cellulitis commonly include coverage for gram-positive and gram-negative microbes. Given this patient's findings and sinusitis also noted on CT imaging, anaerobic coverage was also initiated.

Additionally, in a patient with MRSA bacteremia who develops unilateral periorbital swelling and cannot communicate eye pain or vision changes, it is important that endophthalmitis arising from bacteremic seeding of the eye be considered [20]. A thorough ophthalmology exam is necessary to rule this out since this can be vision-threatening and require intra-vitreous antibiotics to treat [20]. 

In a patient with orbital cellulitis where blood and CSF cultures have no growth and do not aid in the identification of a causative pathogen, empiric coverage based on imaging and common etiologies of orbital cellulitis must guide treatment. Given the severity of orbital cellulitis complications, hospital admission and initiation of intravenous antibiotics with vancomycin, and a third-generation cephalosporin, plus or minus the addition of metronidazole for anaerobic coverage is recommended [10]. Clinical improvement with a decrease in periorbital swelling and resolution of fever is usually achieved within 48 hours of intravenous antibiotics. If no clinical improvement is seen within this time frame and a subperiosteal abscess is present, drainage is considered [10]. In our case, clinical improvement was noted within this time frame and the recommendation from the consulted otolaryngologist was to continue with antibiotic treatment alone, without drainage. 

## Conclusions

This case represents the importance of the physical exam to recognize potential sources of infection and prompt imaging when orbital cellulitis is suspected. In ill-appearing infants presenting with a fever of unknown origin under 60 days old, in addition to the typical laboratory studies, a thorough skin exam is a necessary part of their work-up. Although orbital cellulitis is not common in neonates, clinical signs of periorbital erythema, periorbital swelling, or proptosis should prompt consideration of an orbital cellulitis diagnosis and early imaging to evaluate for intracranial extension or subperiosteal formation should be performed. If orbital cellulitis is diagnosed on imaging, empiric coverage should include coverage for MRSA, gram-negative, and potentially anaerobic bacteria, expanding the typical sepsis rule out antimicrobial coverage. Identification of subperiosteal abscesses should prompt immediate otolaryngologist consultation to determine the need for surgical intervention to reduce the risk of serious complications like vision loss, intracranial abscess development, or meningitis. Ophthalmologist consultation to evaluate endophthalmitis and to perform a thorough ophthalmology examination is also recommended to determine if intra-vitreous antibiotic injections and other vision-saving interventions are needed. 
